# Chloroplast genome features of an important medicinal and edible plant: *Houttuynia cordata* (Saururaceae)

**DOI:** 10.1371/journal.pone.0239823

**Published:** 2020-09-28

**Authors:** Bin Zhu, Qun Feng, Jie Yu, Yu Yu, Xiaoxiang Zhu, Yu Wang, Juan Guo, Xin Hu, Mengxian Cai

**Affiliations:** 1 School of Life Sciences, Guizhou Normal University, Guiyang, People’s Republic of China; 2 The Key Laboratory for Quality Improvement of Agricultural Products of Zhejiang Province, College of Agriculture and Food Science, Zhejiang A&F University, Hangzhou, China; Chinese Academy of Medical Sciences and Peking Union Medical College, CHINA

## Abstract

*Houttuynia cordata* (Saururaceae), an ancient and relic species, has been used as an important medicinal and edible plant in most parts of Asia. However, because of the lack of genome information and reliable molecular markers, studies on its population structure, or phylogenetic relationships with other related species are still rare. Here, we *de novo* assembled the complete chloroplast (cp) genome of *H*. *cordata* using the integration of the long PacBio and short Illumina reads. The cp genome of *H*. *cordata* showed a typical quadripartite cycle of 160,226 bp. This included a pair of inverted repeats (IRa and IRb) of 26,853 bp, separated by a large single-copy (LSC) region of 88,180 bp and a small single-copy (SSC) region of 18,340 bp. A total of 112 unique genes, including 79 protein-coding genes, 29 tRNA genes, and four rRNA genes, were identified in this cp genome. Eighty-one genes were located on the LSC region, 13 genes were located on the SSC region, and 17 two-copy genes were located on the IR region. Additionally, 48 repeat sequences and 86 SSR loci, which can be used as genomic markers for population structure analysis, were also detected. Phylogenetic analysis using 21 cp genomes of the Piperales family demonstrated that *H*. *cordata* had a close relationship with the species within the *Aristolochia* genus. Moreover, the results of mVISTA analysis and comparisons of IR regions demonstrated that the cp genome of *H*. *cordata* was conserved with that of the *Aristolochia* species. Our results provide valuable information for analyzing the genetic diversity and population structure of *H*. *cordata*, which can contribute to further its genetic improvement and breeding.

## Introduction

Piperales, which show discrete vascular bundles in the stem and threefold flower parts [1], are placed in the Magnoliids clade, which is an early evolutionary branch in the angiosperm tree [2]. The category of Piperales has changed in different ways over time. According to the recent APG IV system, Piperales includes three families (Piperaceae, Aristolochiaceae and Saururaceae), 17 genera, and more than 4,000 species, most of which belong to the Piperaceae family [3]. Saururaceae is the smallest family in Piperales, including only four genera and six species, most of which are aromatic herbs with creeping rhizomes [3–5].

*Houttuynia cordata*, the single member of the *Houttuynia* genus in the Saururaceae family, is widely used as a leafy vegetable and medicinal herb throughout much of Asia [6]. Its characteristic extract, houttuynin, has been proven to have diverse pharmacological effects including anticestodal [7], antibacterial [8], and antiviral activity [9]. Moreover, extracts of *H*. *cordata* have had important functions in improving the immune system of patients with severe acute respiratory syndrome (SARS) [10]. Although *H*. *cordata* is the only species in the *Houttuynia* genus of the Saururaceae family, its populations from different regions vary widely at chromosome numbers (2n = 24–128) and polyploidy levels [6, 11]. This probably resulted from the prevailing cytomixis and meiotic abnormalities that occur during microsporogenesis [11]. Almost all previous studies on this plant have focused mainly on its physiological and biochemical properties [7, 9, 10, 12, 13], whereas few studies were aimed at deciphering the genetic diversity, population structure, and taxonomic status of *H*. *cordata* in Piperales. This is mostly likely due to the limited genome information and development of markers.

Chloroplasts have their own circular genome and play a vital role in photosynthesis, physiology and development in most plants. For most angiosperms, chloroplast (cp) genomes typically range in size from 120 to 170 kilobase pairs (kb) [14]. Compared to the nuclear genome, the cp genome is more conserved in terms of gene size and content, genome structure, and linear order of the genes [15]. Generally, the cp genome has a quadripartite cycle, comprising a pair of inverted repeat regions (IRA and IRB) that are separated by one large single-copy (LSC) region and one small single-copy (SSC) region. Previous studies have demonstrated that, compared to the nuclear genome, fewer substitutions of nucleotides and rearrangements of genome structure occur in the cp genome [16, 17], making it an ideal model to decipher genome evolution and phylogenetic relationships in complex angiosperm families [18, 19]. The development of next-generation sequencing technologies has provided highly efficient, low-cost DNA sequencing platforms that produce large volumes of short reads [20]. Moreover, the third-generation sequencing technology, PacBio sequencing platform, otherwise called single-molecule teal-time (SMRT) sequencing technology, generates long reads with lengths of up to 30 kb that can easily close the gaps in current reference assemblies through extended repetitive regions. Already, thousands of cp genomes have been completely revealed. Recently, two short communications have reported the length and gene contents of cp genomes in *H*. *cordata* [21, 22]. However, a comprehensive analysis of this cp genome has not yet been performed.

In the present study, we *de novo* assembled the complete cp genome of *H*. *cordata*, a quadripartite cycle of 160,226 bp, through a combination of PacBio and Illumina sequencing platforms. The rates of synonymous (Ks) and non-synonymous (Ka) substitutions for shared common genes between *H*. *cordata* and related species were also calculated. Phylogenetic analysis using 21 Piperales species demonstrated that *H*. *cordata* had a close relationship with the species within the *Aristolochia* genus. Our results provide valuable information for future research in genetic improvement, population genetics, and population diversity of *H*. *cordata*.

## Materials and methods

### Plant materials and sequencing

The clones of *H*. *cordata* were collected from Guiyang Huaxi Wetland Park, China, and were grown in a glasshouse at Guizhou Normal University. The intact chloroplasts of *H*. *cordata* were initially enriched from 5 g of fresh leaves as described by Okegawa and Motohashi [23] with minor modifications. Briefly, 5 g fresh leaves excluding petioles and veins were homogenized in 400 mM sorbitol, 5 mM MgCl_2_, 5 mM MnCl_2_, 2 mM EDTA, 10 mM NaHCO3, 0.5% (w/v) bovine serum albumin (BSA), 5 mM ascorbate, and 20 mM Tricine–NaOH (pH 8.4). After centrifugation at 3000 g for 5 min, the pellet was gently suspended in 400 mM sorbitol, 5 mM MgCl_2_, 2.5 mM EDTA, and 50 mM 4-(2-hydroxyethyl)-1-piperazineethanesulfonic acid (HEPES)–KOH (pH 7.6). The intact chloroplasts were further purified using 40% (v/v) Percoll (Zhuoyue, Beijing Baiaolaibo, China, http://www.baiaolaibo.com/). Isolated intact chloroplasts were suspended in 3 mM MgCl_2_ and 25 mM HEPES–KOH (pH 7.6), followed by centrifugation at 10,000 g for 3 min. Then, the cp DNA was isolated from the chloroplasts using the DNeasy Plant Mini Kit (Qiagen, USA) according to the manufacturer’s instructions. After assessing the quality and quantity of the cp DNA, one DNA library with 20 kb insertion using the PacBio Sequel platform (PacBio, USA) and one DNA library with 450 bp insertion using the Illumina HiSeq X Ten platform (Illumina, USA) were sequenced based on the manufacturer’s instructions. All the raw data, including short Illumina reads and long PacBio reads used in this study are available at the BIG Sub (http://bigd.big.ac.cn), under the accession number: CRA002843.

### Genome assembly and gene annotation

The 150 bp paired-end reads were generated by the Illumina HiSeq platform. After removing sequencing adapters and low-quality reads with QC values less than 20%, the clean reads were firstly compared to the complete cp genome of the related *Aristolochia tubiflora* (GenBank accession: NC_041455) using BLASTn software [24] (E-value: 10–6) to select cp genome sequences. These selected Illumina reads were then *de novo* assembled into scaffolds using SOAP denovo v2.04 (http://soap.genomics.org.cn/soapdenovo.html) with a K-mer value of 51. Low-quality PacBio reads (minimum read length of 500 bp and minimum read quality of 0.80), were removed from the raw data. Then the filtered PacBio reads were error-corrected to remove single nucleotide insertion/deletions using Illumina short reads by the PacBioToCA module of the Celera Assembler with default parameter settings (-length 500, -partitions 200) [25]. Afterwards, the corrected PacBio reads were used to gap-fill the scaffolds by PBjelly (https://sourceforge.net/projects/pb-jelly/) [26] with all PacBio reads >8kb to generate a circular cp genome map. Frame-shift errors were manually corrected during gene prediction.

The protein-coding genes and noncoding genes (tRNAs and rRNAs) of *H*. *cordata* cp genome were annotated using Dual Organellar GenoMe Annotator (DOGMA, http://dogma.ccbb.utexas.edu/) with default parameters [27]. Moreover, the intron and exon boundaries of protein-coding genes were manually modified. Similarly, the tRNA genes were verified using the tRNAscan-SE 1.23 program (http://lowelab.ucsc.edu/tRNAscan-SE/). The circular gene map of *H*. *cordata* cp genome was given by OGDraw v1.2 [28]. Finally, the complete *H*. *cordata* cp genome was deposited into GenBank (accession number: MN413197).

### Repeat sequence and simple sequence repeat detection

The web service of REPuter (https://bibiserv.cebitec.uni-bielefeld.de/reputer/, [29]) was employed to identify repeat sequences according to the following parameters: minimal repeat size to 30; maximum computed repeats to 50; and hamming distance to 10. Match direction included forward, palindrome, reverse, and complement repeat types. To detect simple sequence repeats (SSRs), MISA (https://webblast.ipk-gatersleben.de/misa/, [30]) was used with minimal repeat numbers set at ten, six, four, three, three, and three for mono-, di-, tri-, tetra-, tetra-, penta-, and hexa-nucleotides, respectively.

### Codon usage analysis

Codon usage bias is a universal feature of all genomes and has been proposed to regulate translation dynamics such as translation efficiency and accuracy, as well as protein folding [31]. To further analyze *H*. *cordata* cp genome evolution, the CodonW1.4.2 program (http://downloads.fyxm.net/CodonW-76666.html) was employed to calculate the synonymous codon usage of protein-coding genes with default settings.

### Phylogenetic analysis

The 20 complete cp genomes (S1 Table) from Aristolochiaceae and Piperaceae families downloaded from NCBI together with *H*. *cordata* cp genome were used to construct their phylogenetic relationship. Only the homologous CDs were used to construct the phylogenetic relationship to reduce data redundancy, such as that done in the study of *Rhododendron delavayi* [32]. A total of 75 homologous CDs (coding gene sequence) were employed in this study, including *psbB*, *psbZ*, *ndhF*, *petG*, *rps18*, *rpoB*, *petN*, *psbA*, *psaJ*, *ccsA*, *rpoC1*, *rbcL*, *psbM*, *ndhG*, *rps19*, *rpl2*, *psbL*, *psaC*, *rps3*, *matK*, *psbE*, *rpl14*, *petA*, *rpl33*, *psaA*, *rpl36*, *ndhK*, *ndhJ*, *psbK*, *atpI*, *psbF*, *psbI*, *rps2*, *rpl32*, *atpH*, *psbN*, *accD*, *psaB*, *rps11*, *atpA*, *rps14*, *infA*, *psaI*, *ycf1*, *rps4*, *atpE*, *psbJ*, *rpl16*, *rps15*, *rpl20*, *rpoA*, *ndhD*, *psbH*, *rpoC2*, *ndhI*, *ndhA*, *ndhH*, *rps16*, *atpB*, *petD*, *psbC*, *ndhB*, *petB*, *atpF*, *rps7*, *rpl23*, *psbD*, *clpP*, *rpl22*, *ndhE*, *petL*, *psbT*, *ycf2*, *rps12*, and *ndhC*, were used to determine the phylogenetic relationship. Phylogenetic trees were then constructed using the maximum likelihood (ML) method with 1000 bootstrap replicates by MEGA7 [33].

### Whole cp genome sequence comparisons

To provide comprehensive knowledge of cp sequence divergence, the *H*. *cordata* cp genome was compared to four cp genomes from the *Aristolochia* genus. The divergence of the LSC/IRB/SSC/IRA boundary regions among these related species was visualized by IRscope (https://irscope.shinyapps.io/irapp/) based on the annotations of their available cp genomes in GenBank. Additionally, the mVISTA program (http://genome.lbl.gov/vista/mvista/submit.shtml) was also used to compare the whole cp genome divergence among these related species.

### Synonymous and nonsynonymous substitution rate calculations

To determine synonymous (Ks) and non-synonymous (Ka) substitution rates, we performed pairwise comparisons of the 79 protein-coding genes between *H*. *cordata* cp genome and four close Aristolochia species. Pairwise alignments of the common genes among species were carried out by MAFFT [34], and the Ka/Ks ratios were calculated with KaKs_calculator 2.0 [35] using the default parameters for plant plastid code.

## Results

### Chloroplast genome assembly and genome features

The Illumina sequencing platform gave rise to 3,763 Mb of raw data. After trimming, 3,390 Mb of clean reads with a Q20 value of 95.59% was obtained. The PacBio platform generated 137,091 subreads with an average length of 4,871 bp and an N50 length of 7,337 bp (S2 Table). Both Illumina reads and PacBio subreads were used to construct the complete cp genomes of *H*. *cordata*.

**Fig 1:**
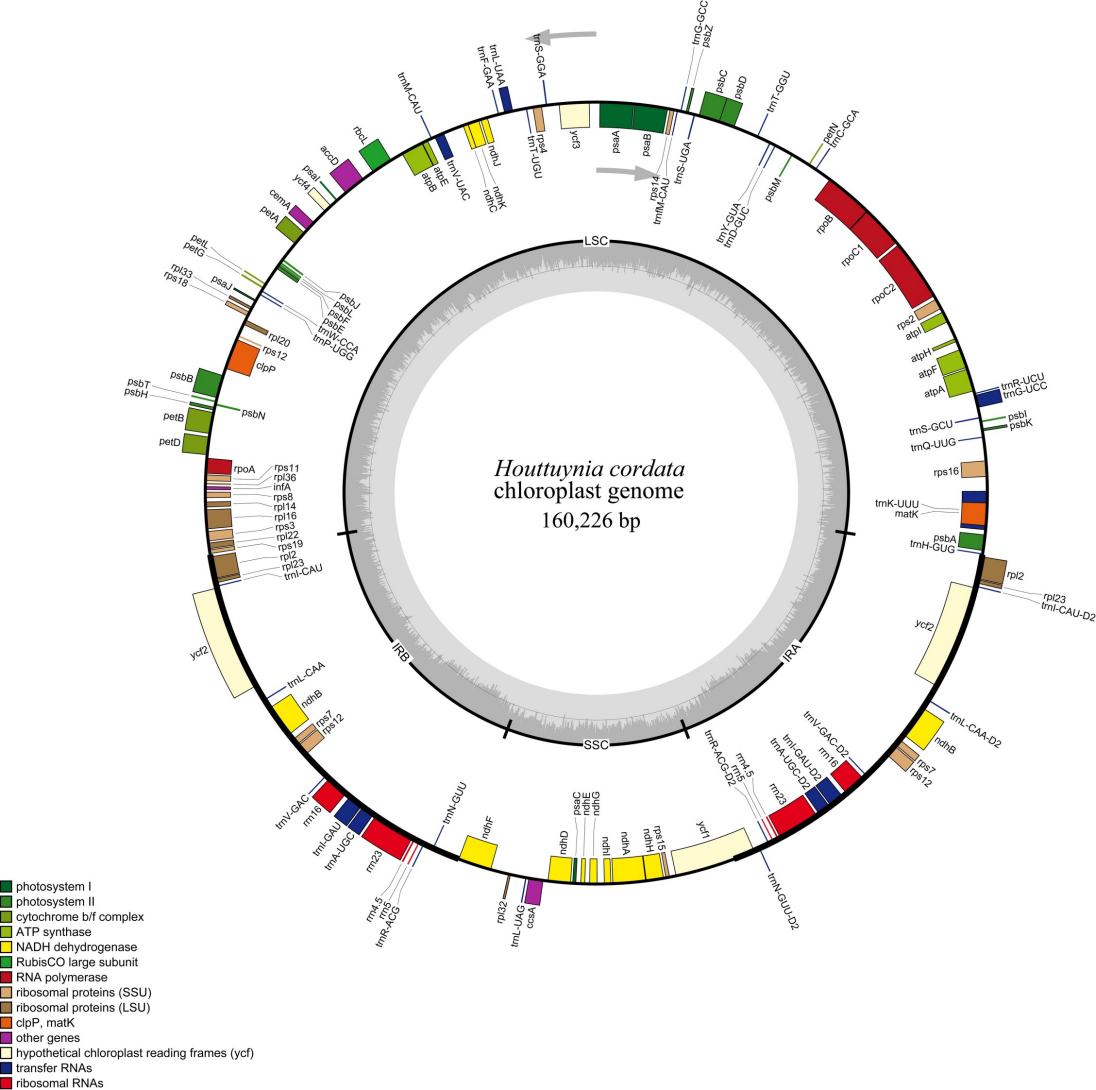
Gene circular map of the complete *H*. *cordata* cp genome. Genes on the outside and inside of the circle are transcribed in clockwise and counterclockwise directions, respectively. Genes belonging to different functional groups are color coded. Color intensity corresponds to GC content. The SSC, LSC, and inverted repeat regions (IRA and IRB) are indicated.

The *H*. *cordata* cp genome showed a typical quadripartite cycle of 160,226 bp, comprising a pair of inverted repeats (IRa and IRb) of 26,853 bp, which were divided by a large single-copy (LSC) region of 88,180 bp and a small single-copy (SSC) region of 18,340 bp (Table 1). Regarding GC content, the IR regions showed the highest GC content of 43.03%, followed by 36.81% in the LSC region, whereas the SSC region exhibited the lowest GC content of 32.15%. The overall GC content of the cp genome was 38.36%. In total, 112 unique genes were identified in the *H*. *cordata* cp genome, including 79 protein-coding genes, 29 tRNA genes, and four rRNA genes (Table 1). Furthermore, out of 112 unique genes, 82 and 13 genes were found in the LSC and SSC regions, respectively; while 17 genes, including six protein-coding genes, seven tRNAs, and four rRNAs were duplicated in the IR regions (Fig 1). Among the 112 unique genes, 18 genes (comprosing 11 protein-coding genes and seven tRNA genes) had one intron, and only two genes (*clpP* and *ycf3*) harbored two introns (Table 2).

**Table 1:** The detail features of the complete cp genome of *H*. *cordata*.

Category	Items	Descriptions
Construction of cp genome	LSC region (bp)	88,180
	IRA region (bp)	26,853
	SSC region (bp)	18,340
	IRB region (bp)	26,853
	Genome Size (bp)	160,226
Gene content	Unique genes	112
	Protein-coding genes	79
	tRNAs	29
	rRNAs	4
	Two copy genes	17
	Genes on LSC region	82
	Genes on IRA region	17
	Genes on SSC region	13
	Genes on IRB region	17
	Gene total length (bp)	160,226
	Average of genes length (bp)	926
	Gene length/Genome (%)	49.13
GC content	GC content of LSC region (%)	36.81
	GC content of IRA region (%)	43.03
	GC content of SSC region (%)	32.15
	GC content of IRB region (%)	43.03
	Overall GC content (%)	38.36

**Table 2:** Summary of assembled gene functions of *Houttuynia cordata* Thunb cp genome.

Category for genes	Genes function	Genes
**Genes involving in photosynthesis**	Subunits of photosystem	*psaA*, *psaB*, *psaC*, *psal*, *psaJ*, *psbA*, *psbB*, *psbC*, *psbD*, *psbE*, *psbF*, *psbH*, *psbI*, *psbJ*, *psbK*, *psbL*, *psbM*, *psbN*, *psbZ*, *psbT*,
	Subunits of cytochrome b/f complex	*petA*, *petB*^*b*^, *petD*^*b*^, *petG*, *petL*, *petN*
	Large subunit of Rubisco	*rbcL*
	Subunits of ATP synthase	*atpA*, *atpB*, *atpF*^*b*^, *atpH*, *atpI*, *atpE*
	Subunits of NADH-dehydrogenase	*ndhA*^*b*^, *ndhB*^*a*^^,^^*b*^, *ndhC*, *ndhD*, *ndhE*, *ndhF*, *ndhGndhH*, *ndhI*, *ndhJ*, *ndhK*,
**Self-replication**	Ribosomal RNA genes	*rrn5*^*a*^, *rrn4*.*5*^*a*^, *rrn23*^*a*^, *rrn16*^*a*^
	Transfer RNA genes	*trnL-CAA*^*a*^, *trnL-UAG*, *trnP-UGG*, *trnW-CCA*, *trnM-CAU*, *trnV-UAC*^*b*^, *trnF-GAA*, *trnL-UAA*^*b*^, *trnT-UGU*, *trnS-GGA*, *trnfM-CAU*, *trnG-GCC*, *trnS-UGA*, *trnT-GGU*, *trnY-GUA*, *trnD-GUC*, *trnC-GCA*, *trnR-UCU*, *trnG-UCC*^*b*^, *trnS-GCU*, *trnQ-UUG*, *trnK-UUU*^*b*^, *trnH-GUG*, *trnI-CAU*^*a*^, *trnV-GAC*^*a*^, *trnI-GAU*^*a*^^,^^*b*^, *trnA-UGC*^*a*^^,^^*b*^, *trnR-ACG*^*a*^, *trnN-GUU*^*a*^
	Small subunit of ribosome	*rps11*, *rps12*^*a*^^,^^*b*^, *rps14*, *rps15*, *rps16*^*b*^, *rps18*, *rps19*, *rps2*, *rps3*, *rps4*, *rps8*, *rps7*^*a*^
	Large subunit of ribosome	*rpl2*^*a*^^,^^*b*^, *rpl14*, *rpl16*^*b*^, *rpl20*^*b*^, *rpl22*, *rpl23*^*a*^, *rpl32*, *rpl33*, *rpl36*
	DNA-dependent RNA polymerase	*rpoA*, *rpoB*, *rpoC1b*, *rpoC2*
**Other genes**	Maturase	*matK*
	Envelope membrane protein	*cemA*
	Subunit of acetyl-CoA	*accD*
	C-type cytochrome synthesis gene	*ccsA*
	Translational initiation factor	*infA*
	Protease	*clpP*^*c*^
** Functionally unknown genes**	Conserved open reading frames	*ycf1*, *ycf2*^*a*^, *ycf3*^*c*^, *ycf4*

^a,b,c^The letters indicate the gene with two copes, harboring one intron and two introns, respectively.

### Repeat sequence and Simple Sequence Repeat (SSR) detection

**Fig 2:**
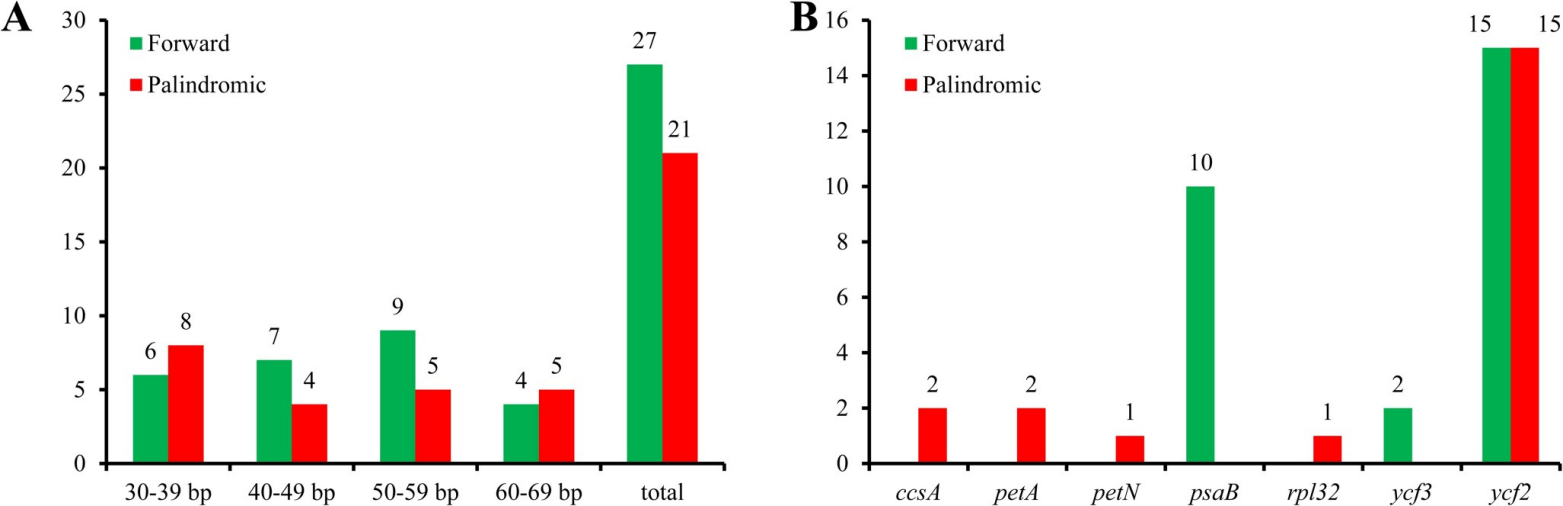
Distribution of repeat sequences in *H*. *cordata* cp genome. A, Number of forward and palindromic types of repeats with different size in length; B, Number of repeats within seven genes.

In this study, a total of 48 repeat sequences with lengths ranging from 30 bp to 69 bp were detected, including 27 forward repeats and 21 palindromic repeats, whereas no reverse or complement repeats were identified (S3 Table; Fig 2A). Among these repeats, 14 were 30–39 bp in length, 11 were 40–49 bp, 14 were 50–59 bp, and nine were 60–69 bp (Fig 2A). All the repeats were found within seven protein-coding genes (*ccsA*, *petA*, *petN*, *psaB*, *rpl32*, *ycf2*, and *ycf3*) (Fig 2B). Out of 30 repeats (62.5%), 15 forward repeats and 15 palindromic repeats, were contributed by *ycf2*, which is essentially a pseudogene. Most paired repeats (36 repeats, 75%) were located in the same genes; however, 12 repeats, that were all forward types, were seen in different genes (S3 Table).

In total, 86 SSR loci of 17 different types with a length of at least 10 bp were also detected by MISA (Table 3). Among the SSR loci, mononucleotide repeats were the most abundant with 68 SSR motifs (79.07%) of only two types (A/T). Eleven tetranucleotide repeats representing 10 different types and four trinucleotide repeats representing three different types (TTA/TAT/ATA) were identified. The dinucleotide repeats were only TA type with two motifs, and the pentanucleotide repeats (TCTTT) was observed only once. No hexanucleotide repeats were observed, and the longest SSR was a tetranucleotide repeat (TTTA) of 24 bp in length.

**Table 3:** Summary of simple sequence repeats in *Houttuynia cordata* cp genome.

Repeats Unit	Types	Number	Largest Repeats (bp)
1	T	40	15
	A	28	15
2	TA	2	16
3	TTA	1	12
	TAT	1	12
	ATA	2	12
4	AATG	1	12
	ATCT	1	12
	ATTA	1	12
	CAAA	1	12
	CATT	1	12
	CACT	1	12
	TATG	1	12
	TCAA	1	12
	TTTA	2	24
	TTTC	1	12
5	TCTTT	1	15
Total	17	86	—

### Codon usage analysis

**Fig 3:**
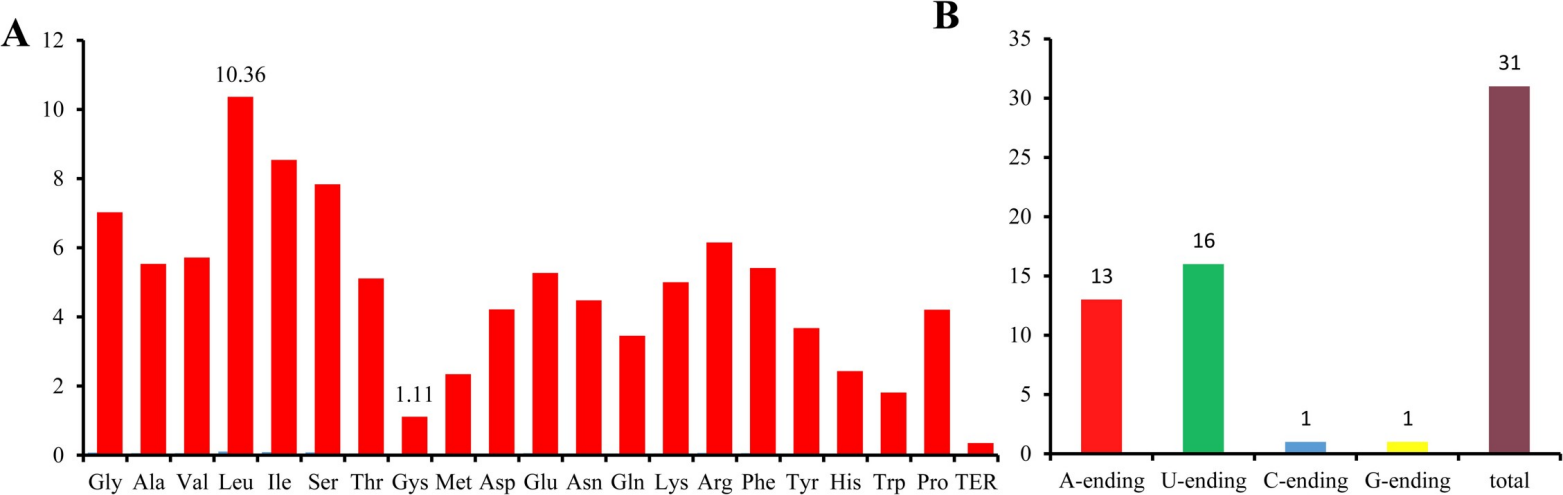
Percentage of amino acids of *H*. *cordata* cp genome and the ending patterns of biased usage codons (RSUC>1). A, Detail information of amino acids used in *H*. *cordata* cp genome. B, The A- and U- ending patterns were the most popular in biased usage codons.

The coding sequence of 79 protein-coding genes gave rise to 22,816 codons. Among these, the leucine codons were the most biased, with a frequency of 10.36%, whereas the cysteine codons had the lowest usage frequency of only 1.11% (S4 Table; Fig 3A). To gain knowledge of synonymous codon usage bias of *H*. *cordata* cp genome, we also calculated the relative synonymous codon usage (RSCU) value. The results showed that the RSCU values of 31 codons were greater than 1, indicating these codons were preferentially used. Among these preferential codons, the majority of codons ended with A (13 of 31) or U (16 of 31) except UUG and UCC (S4 Table; Fig 3B).

### Phylogenetic analysis and whole cp genome sequence comparisons

**Fig 4:**
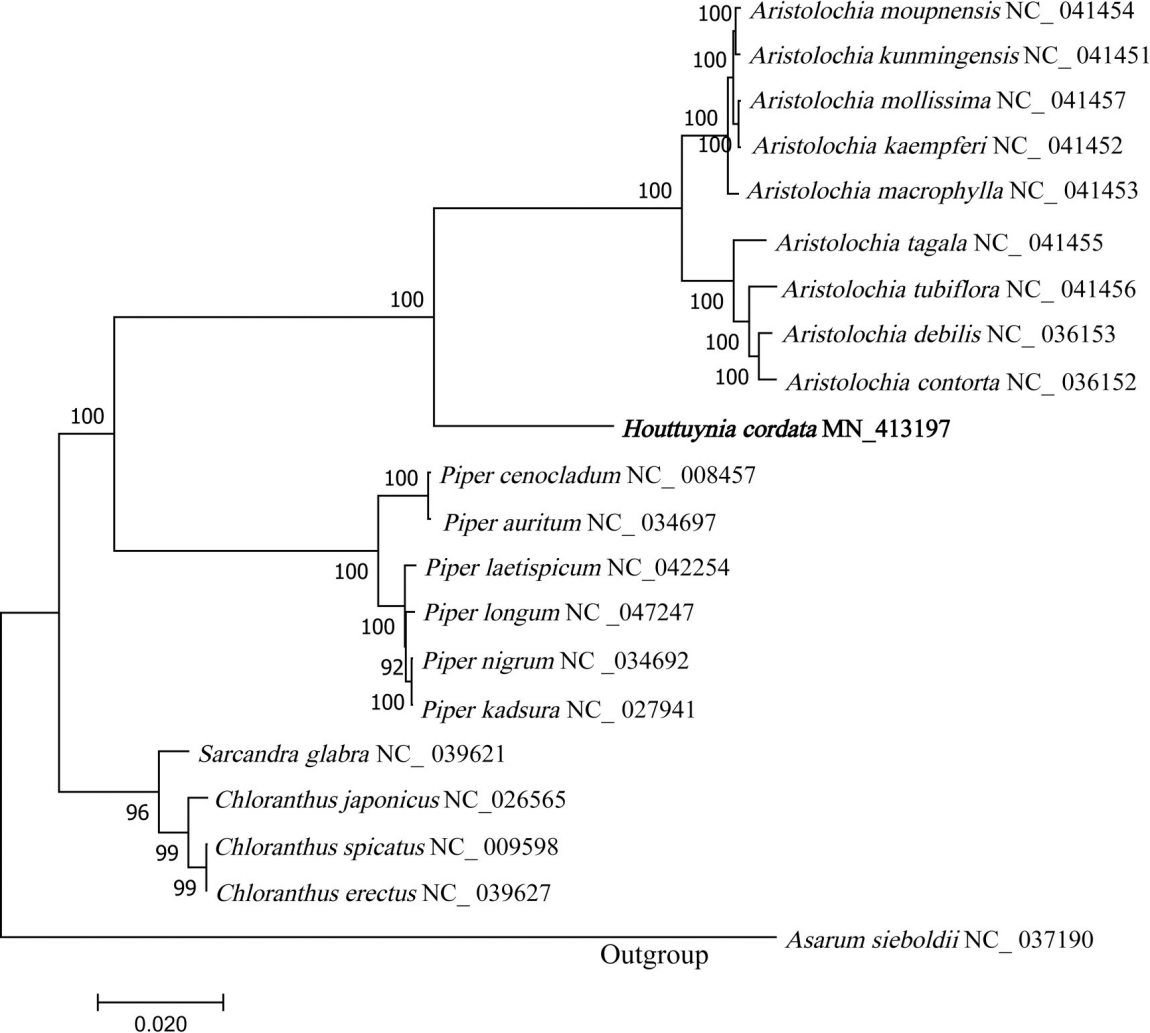
Phylogenetic relationship analysis of 21 Piperales species based on the shared common protein-coding sequence. The evolutionary history was inferred using the Maximum Likelihood method based on the Tamura-Nei model. The bootstrap values are shown next to the branches. The initial tree(s) for the heuristic search were obtained automatically by applying the Neighbor-Join and BioNJ algorithms to a matrix of pairwise distances estimated by the Maximum Composite Likelihood (MCL) approach, and then selecting the topology with the highest log value. The tree is drawn to scale, with branch length measured by the number of substitutions per site.

To analyze phylogenetic relationships among the Piperales family, we downloaded 20 complete cp genomes covering four genera (S1 Table) from NCBI to construct the phylogenetic trees. To reduce data redundancy, 75 homologous CDs of all 21 cp genomes were used to generate phylogenetic tree by the Maximum Likelihood method with 1000 bootstrap replicates. The phylogenetic tree generated 19 nodes, and *Asarum sieboldii* (NC_037190) formed the outgroup. Out of 19 nodes, 18 nodes bootstrap values were ≥92%. Generally, the phylogenetic tree showed that each genus (*Aristolochia*, *Piper*, and *Chloranthus*) constituted a monophyletic group. *H*. *cordata* together with the species of *Aristolochia* formed a subgroup with bootstrap values of 100%, indicating that they shared a more closer relationship (Fig 4).

**Fig 5:**
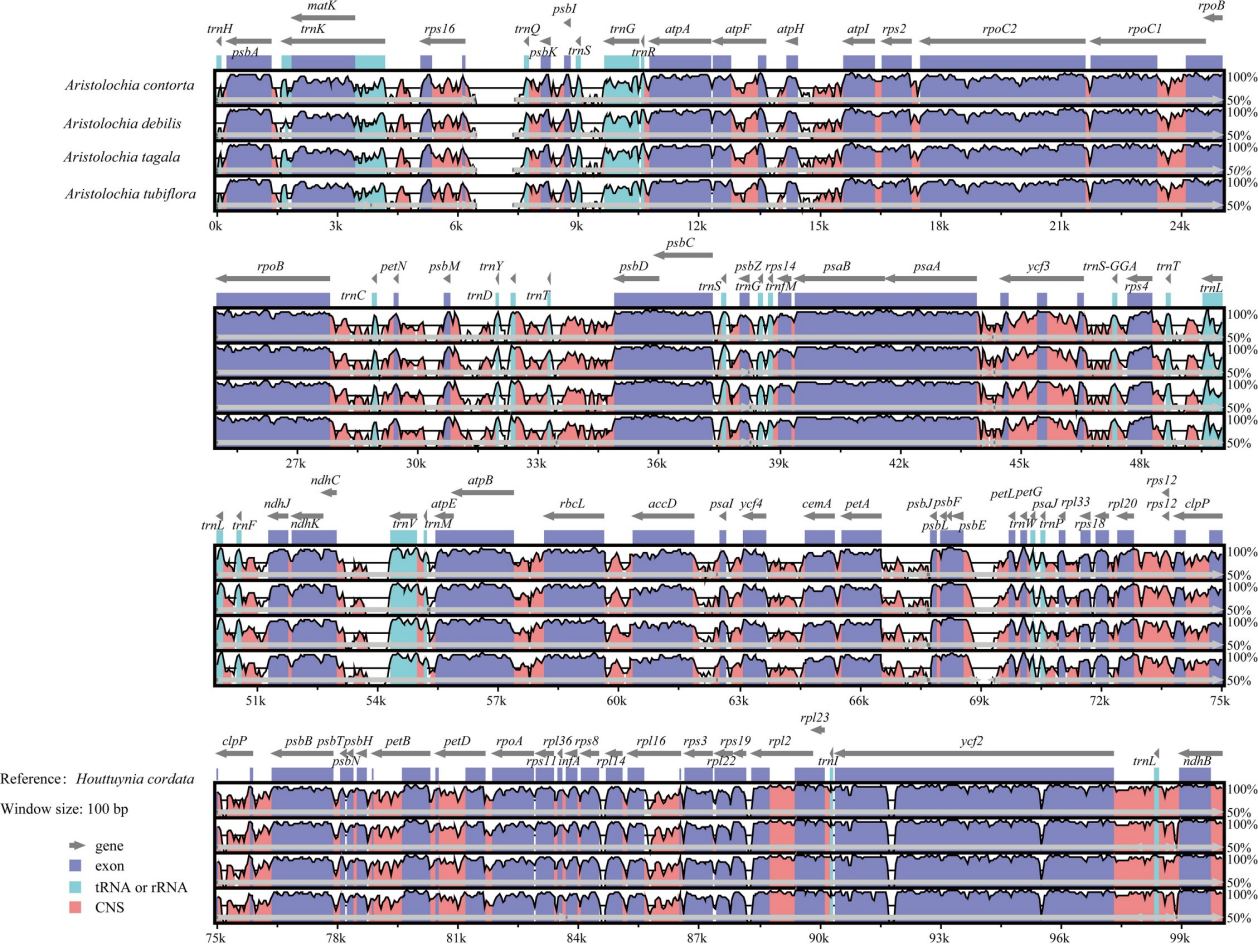
Alignment of the cp genomes of *H*. *cordata* and four closely related species. The alignment was performed by mVISTA with *H*. *cordata* as a reference. Local collinear blocks within each alignment are indicated by the same color and linked.

Additionally, mVISTA analysis was performed to evaluate divergence in the genome sequences between *H*. *cordata* and *Aristolochia* species with reference to the annotation of *H*. *cordata*. The results revealed that all selected cp genomes showed generally high similarity (>85%). However, several minor inserted regions and one large inserted region beyond 1 kb were observed (Fig 5), indicating that the *H*. *cordata* cp genome underwent evolutionary divergence. Overall, the coding regions were more conserved than the non-coding regions and the obviously divergent genes were *ycf2*, *rpl14*, *rpl19*, *atpH*, and *rpl22*.

**Fig 6:**
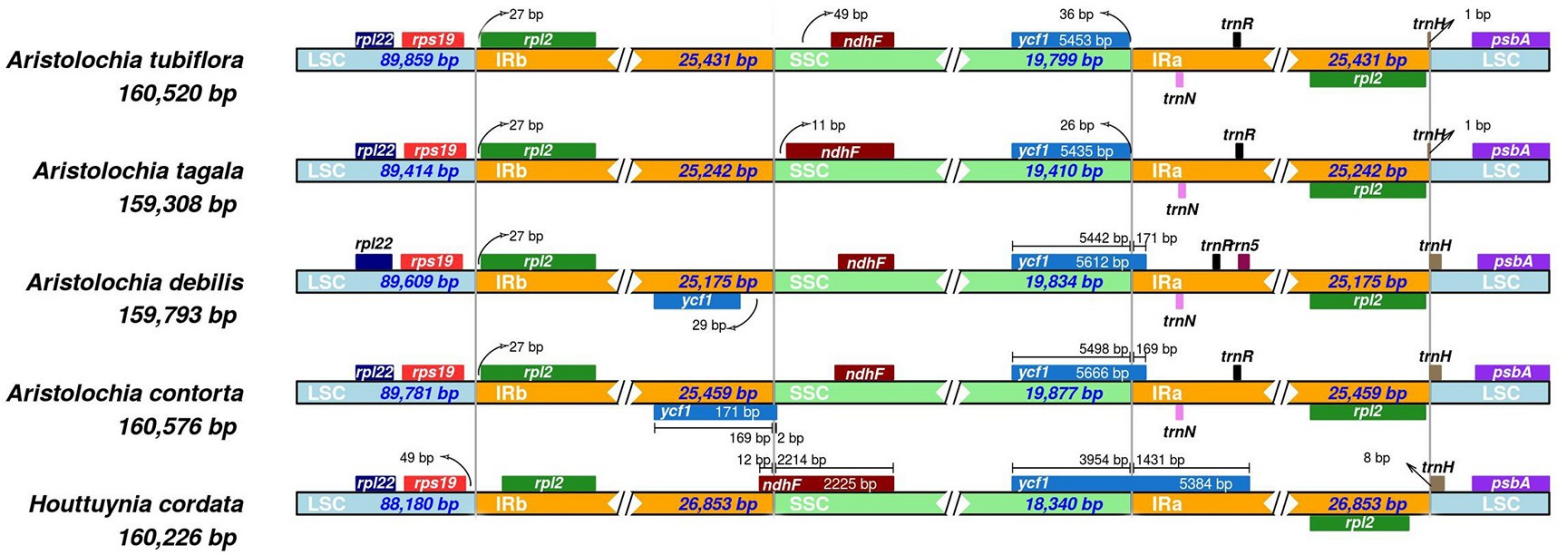
Analysis of the boundaries of LSC/SSC/IR and adjacent genes among five Piperales cp genomes. Sequences of the LSC/IRB/SSC/IRA boundaries and adjacent genes in *H*. *cordata* and four closely related cp genomes, including *A*. *contorta*, *A*. *debilis*, *A*. *tagala*, and *A*. *tubiflora*, were downloaded from GenBank and analyzed.

To gain a more comprehensive comparison, the IR regions that were believed to contribute to the size variation of the cp genome were also compared among these five species (Fig 6). The results showed that, compared to IRB/SSC and SSC/IRA junctions, the distribution of the genes in the LSC/IRB and IRA/LSC border regions were relatively conserved. The *rps19* and *rpl2*, and *trnH* genes were distributed at the LSC/IRB and IRA/LSC junction in all five cp genomes, differing only in the distance of three genes to the junction. For SSC/IRA boundaries, the *ycf1* gene located on the SSC region had a 169 bp, 171 bp, and 1431 bp extension to the IRA region in *A*. *contorta*, *A*. *debilis*, and *H*. *cordata*, respectively. However, a 26 bp and 36 bp distance of the *ycf1* gene to the boundary were found in *A*. *tagala* and *A*. *tubiflora*, respectively. For IRB/SSC boundaries, the *ndhF* gene located on the SSC region was either near or overlapping the boundary in *A*. *tagala*, *A*. *tubiflora*, and *H*. *cordata*, whereas in *A*. *contorta* and *A*. *debilis*, the gene was replaced by the *ycf1* gene located on IRB.

### Synonymous (Ks) and non-synonymous (Ka) substitutions rate analysis

The Ka/Ks ratio has been considered as well-recognized marker for assessing genome evolution and selection pressure affecting genes [36, 37]. The Ka/Ks ratio of the pairwise common 79 protein-coding genes between *H*. *cordata* cp genome and four related *Aristolochia* species was calculated (S5 Table). All Ka/Ks values of common genes were below 1 in all four comparisons. Of 79 homologous CDs, 76 CDs (96.20%) had a Ka/Ks value below 0.5 in all tested comparisons, and only the *ycf2* gene always had a Ka/Ks value above 0.5. Overall, the average Ka/Ks value of all common genes was 0.149. Additionally, 31 common genes (44.93%) had an average Ka/Ks value below 0.1, suggesting that these genes have undergone strong purifying selection pressures in *H*. *cordata* cp genome.

## Discussion

*H*. *cordata*, known for its use as a vegetable and medicinal for functions, is a member of the Saururaceae family, which is believed to be an ancient and relic family [38]. The Saururaceae, Piperaceae, and Aristolochiaceae families together form the Piperales, which is an early diverging lineage [3, 38]. Because of the lack of a related cp genome, we employed the combination of PacBio and Illumina sequencing platforms, an effective strategy for the assembly of cp genome without a reference [39, 40] to *de novo* assemble the *H*. *cordata* cp genome. The complete cp genome of *H*. *cordata* reported here showed a typical quadripartite cycle of 160,226 bp in length, which was consistent with a previous report of cp genome of *H*. *cordata* [22]. However, unlike the previous study, we found one more protein-coding gene and one less tRNA were obtained in the present study. Another short communication also reported a complete cp genome of *H*. *cordata* 161,090 bp in length, harboring 81 protein-coding genes [21]. These studies indicate that the cp genome of *H*. *cordata* has undergone divergence whether it is more or less. Because the two reports could not provide detailed information on gene contents and genome information, the divergent hot spots in cp genome of *H*. *cordata* could not be detected. We also noticed that the cp genome of *H*. *cordata* obtained in the present study was comparable with that of published *Aristolochia* (Aristolochiaceae) species (159,308–160,520 bp; S1 Table) [41, 42], *Piper* (Piperaceae) species (159,909–161,721 bp) and *Saruma henryi* (Aristolochiaceae) (159,914 bp). This finding indicates that cp genomes of the Piperales species were conserved in length. However, the cp genome of *H*. *cordata* was slightly larger than that of *Passiflora edulis* (151,406 bp) at length [43]. Moreover, the gene content between *H*. *cordata* and *P*. *edulis* cp genome was much more divergent, where one genes (*trnE-UUC*) were missing in *H*. *cordata* cp genome, whereas six genes (including *rpl20*, *rpl22*, *rps7*, *rps16*, *infA*, and *accD*), were missing in *P*. *edulis* cp genome. The variable IR region and boundary construction in SSC/IR and LSC/IR have been considered as the main driving force for the length variation of angiosperm cp genomes [44]. As shown in Fig 6, the size of the IR region and the adjacent genes in the boundaries of *H*. *cordata* were similar to those of the four selected *Aristolochia* species. Only minor shifts of these adjacent genes occurred within the boundaries.

Repeat sequences are believed to play an important role in genome rearrangements and sequence variations through illegitimate recombination and slipped-strand mispairing [45, 46]. Forty-eight repeat sequences of 30–69 bp were detected in *H*. *cordata* cp genome. Furthermore, among the reported *Aristolochia* species, 38–138 repeats were identified [41, 42], suggesting that repeat sequences were variable between lineages, which can be used as genomic markers for phylogenetic analysis [42, 47]. Intriguingly, the largely pseudogene, *ycf2* contributed nearly two-thirds (30 of 48) of the repeats. Similar results were observed in the cp genomes of *Haberlea rhodopensis*, *Vernicia fordii* and *Nasturtium officinale* [40, 48, 49], indicating that *ycf2* was the most variable in cp genome. A total of 86 SSR motifs of 17 different types were observed in *H*. *cordata* cp genome, fewer than those of the reported *Aristolochia* species (ranging from 95 to 156) [41, 42]. Among these SSRs, mononucleotide repeats with the A/T type were the largest in number, similar to the results of previous studies [40–42, 50, 51]. This is most likely due to high proportions of polyadenine (polyA) and polythymine (polyT) in the cp genome. These results suggest that SSRs reshape the cp genome and are powerful tools for identifying the genetic diversity among different species.

## Conclusion

Herein, the complete *H*. *cordata* cp genome was *de novo* assembled by integrating the Illumina and PacBio platforms. The *H*. *cordata* cp genome showed a typical quadripartite cycle of 160,226 bp, which comprised 79 protein-coding genes, 29 tRNA genes, and four rRNA genes. A total of 48 repeat sequences and 86 SSR loci were identified, which could be used for marker development as well as phylogenetic and population studies in *H*. *cordata*. Moreover, codon usage analysis revealed that the Leu codon ending with A/U was preferentially utilized. The phylogenetic tree of 21 Piperales species, constructed based on homologous protein-coding genes, demonstrated that *H*. *cordata* had a close relationship with *Aristolochia* species. In summary, this research lays a foundation for future phylogenetic studies on Piperales species and provides useful information for the genetic improvement and breeding of *H*. *cordata*.

## Supporting information

S1 Table List of the cp genome of 16 Piperales species used for phylogenetic analysis. (XLSX)

S2 Table Summary of de novo sequencing of cp genome of *H*. *cordata* Thunb. (XLSX)

S3 Table Detail information of repeat sequence of *H*. *cordata* cp genome. (XLSX)

S4 Table Codon usage analysis of protein coding genes of *H*. *cordata* cp genome. (XLSX)

S5 Table Synonymous (Ks) and non synonymous (Ks) substitution rate of homologous coding genes between *H*. *cordata* and other four closed related species. (XLSX)

## Abbreviations

Cp: chloroplast
IR: inverted repeat
LSC: large single copy
SSC: small single copy
rRNA: ribosomal RNA
tRNA: transfer RNA
SSR: simple sequence repeat
RSCU: the relative synonymous codon usage
ML: maximum likelihood
Ka: non-synonymous
Ks: synonymous
